# 伴NPM1突变急性髓系白血病患者早期死亡的危险因素分析

**DOI:** 10.3760/cma.j.cn121090-20250929-00452

**Published:** 2026-04

**Authors:** 淋淋 王, 铭锴 舒, 雪晴 窦, 亚男 马, 睿 姜, 会慧 陈, 月新 程, 苏宁 陈

**Affiliations:** 1 盐城市第一人民医院，徐州医科大学盐城临床学院，南京大学医学院附属盐城第一医院，盐城 224000 Department of Hematology, the First People's Hospital of Yancheng, Affiliated Hospital of Nanjing University Medical School, the Yancheng Clinical College of Xuzhou Medical University, Yancheng 224000, China; 2 苏州大学附属第一医院，江苏省血液研究所，国家血液系统疾病临床医学研究中心，苏州 215006 National Clinical Research Center for Hematologic Diseases, Jiangsu Institute of Hematology, the First Affiliated Hospital of Soochow University, Suzhou 215006, China

**Keywords:** 白血病，髓系，急性, NPM1突变, 早期死亡, CD34和HLA-DR双阴性表型, Leukemia, myeloid, acute, NPM1 mutation, Early death, CD34⁻/HLA-DR⁻ phenotype

## Abstract

**目的:**

探讨伴NPM1突变急性髓系白血病（NPM1^mut^ AML）患者早期死亡（ED）的危险因素。

**方法:**

回顾性分析2018年6月至2023年6月苏州大学附属第一医院和盐城市第一人民医院收治的初诊原发性NPM1^mut^ AML患者共405例，对患者诊断时的临床和实验室资料进行分析。应用单因素及多因素Logistic回归分析方法探讨NPM1^mut^ AML患者ED的影响因素。

**结果:**

405例NPM1^mut^ AML患者的中位年龄为53（14～86）岁，男177例，女228例，诊断时常见的突变为DNMT3A（51.8％）、FLT3-ITD（51.3％）、FLT3-TKD（25.1％）、IDH2（21.5％）和TET2（21.0％）突变*。*ED患者35例（8.6％），其中死于重症感染14例（40.0％）、脑出血11例（31.4％）、脑梗死4例（11.4％）、肺出血3例（8.6％）。与非ED患者相比，ED组患者CD34⁻/HLA-DR⁻免疫表型的比例较高（71.4％对30.0％，*P*<0.001），凝血功能异常发生率较高（80.0％对27.5％，*P*<0.001），TET2突变发生率较高（40.0％对19.1％，*P*＝0.004）。多因素分析显示查尔森合并症指数（CCI）（*OR*＝10.540，*P*＝0.003）、低血小板计数（*OR*＝10.980，*P*＝0.001）、凝血功能异常（*OR*＝7.126，*P*＝0.005）、CD34⁻/HLA-DR⁻（*OR*＝6.416，*P*＝0.002）和重症感染（*OR*＝59.080，*P*<0.001）是患者ED的独立危险因素。

**结论:**

重症感染和出血是NPM1^mut^ AML患者ED的主要危险因素，CD34⁻/HLA-DR⁻免疫表型增加了NPM1^mut^ AML患者的ED风险，应纳入临床管理中，以指导早期临床干预。

## Abstract (en)

**Objective:**

To investigate the risk factors for early death（ED）in patients with NPM1-mutated acute myeloid leukemia（NPM1^mut^ AML）.

**Methods:**

A retrospective analysis was conducted on 405 newly diagnosed primary NPM1^mut^ AML patients admitted to the First Affiliated Hospital of Soochow University and the First People's Hospital of Yancheng between June 2018 and June 2023. Clinical and laboratory data at diagnosis were analyzed. Univariate and multivariate logistic regression analyses were used to explore the risk factors for early death in these patients.

**Results:**

The median age of the 405 NPM1^mut^ AML patients was 53 (14–86) years, with 177 males and 228 females. The most common mutations at diagnosis were in DNMT3A（51.8％）, FLT3-ITD（51.3％）, FLT3-TKD（25.1％）, IDH2（21.5％）, and TET2（21.0％）. Thirty-five patients (8.6％）experienced ED, with causes including 14 cases（40.0％）of severe infection, 11 cases（31.4％）of cerebral hemorrhage, 4 cases（11.4％）of cerebral infarction, and 3 cases（8.6％）of pulmonary hemorrhage. Compared with non-ED patients, ED patients had a significantly higher proportion of the CD34 and HLA-DR double-negative immunophenotype（CD34⁻/HLA-DR⁻）（71.4％ *vs* 30.0％, *P*<0.001）, a higher incidence of coagulopathy（80.0％ *vs* 27.5％, *P*<0.001）, and a higher incidence of TET2 mutations（40.0％ *vs* 19.1％, *P*＝0.004）. Multivariate analysis showed that the Charlson comorbidity index（CCI）（*OR*＝10.540, *P*＝0.003）, platelet count（*OR*＝10.980, *P*＝0.001）, coagulopathy（*OR*＝7.126, *P*＝0.005), CD34⁻/HLA-DR⁻ immunophenotype（*OR*＝6.416, *P*＝0.002）, and infection（*OR*＝59.080, *P*<0.001）were independent risk factors for ED in NPM1^mut^ AML patients.

**Conclusion:**

Severe infection and bleeding were the main risk factors for ED in NPM1^mut^ AML patients, and the CD34⁻/HLA-DR⁻ immunophenotype should be considered in risk stratification and clinical management of patients with NPM1^mut^ AML to guide early intervention.

伴NPM1突变的急性髓系白血病（Acute myeloid leukemia with NPM1 mutation, NPM1^mut^ AML）患者虽然整体预后较好，但结局仍存在较大的异质性。已有大量文献报道一些临床特征及分子学异常与不良预后相关，包括年龄、白细胞计数、NPM1突变类型、FLT3-ITD突变及微小残留病（MRD）[1]–[3]。在欧洲白血病网（ELN）2017和ELN 2022指南中，只有FLT3-ITD突变和不良预后核型用于预后分层[3]–[4]，其他的协同突变，如DNMT3A、IDH1、IDH2、RAS、WT1和MDS相关突变等，对NPM1^mut^ AML患者预后的影响尚不明确[1],[5]–[8]。另外，NPM1突变类型对预后的影响也依然存在争议，有研究者发现NPM1-非ABD型突变与较差的总生存（OS）和MRD阳性相关，并增加了MRD阴性患者的累积复发率（CIR），但同时又有研究发现NPM1-D型突变患者的无事件生存（EFS）和OS较差，且是影响OS的独立危险因素[1],[9]。化疗后的MRD具有较强的预后意义，并影响了患者的预后分层和治疗策略的选择，目前几项大型研究及指南推荐2个疗程后的外周血MRD作为评估预后及移植的预测因素[3]–[4],[10]。此外，治疗强度对NPM1^mut^ AML患者的结局也存在影响，如FLAG方案、吉妥珠单抗和FLT3抑制剂的应用使患者获得了更深的缓解和更好的生存[1],[11]–[13]。

众所周知，AML的早期死亡（Early death, ED）原因常为重症感染、出血、高白细胞淤滞、肿瘤溶解综合征。而NPM1^mut^ AML有一独特的亚型，即“急性早幼粒细胞白血病样（Acute promyelocytic leukemia-like, APL-like）” NPM1^mut^ AML，骨髓细胞形态可见原始细胞中含有大量嗜天青颗粒，流式细胞术免疫表型为CD34和HLA-DR双阴性（CD34⁻/HLA-DR⁻），该亚型患者表现为凝血功能异常，出血事件发生率高[14]–[20]，然而尚未有研究分析NPM1^mut^ AML患者ED的危险因素以及“APL-like”表型与ED的关系。因此，我们对405例原发性NPM1^mut^ AML患者进行回顾性分析，旨在探讨影响该亚组患者ED的危险因素。

## 病例与方法

1. 病例资料：回顾性分析2018年6月至2023年6月在苏州大学附属第一医院和盐城市第一人民医院治疗的405例原发性NPM1^mut^ AML患者（急性早幼粒细胞白血病除外），所有患者符合2022年国际共识分类（ICC）中NPM1^mut^ AML的诊断标准[21]。收集患者的年龄、性别、初诊日期、初诊血常规、乳酸脱氢酶、凝血常规、骨髓细胞形态学、免疫分型、融合基因、染色体和二代基因测序（NGS）结果、治疗方案以及生存情况。

2. ED的定义：从诊断到死亡的时间在30 d以内[22]。

3. 凝血功能异常的定义：凝血酶原时间（PT）较正常值上限>3 s，活化部分凝血活酶时间（APTT）较正常值上限>10 s，纤维蛋白原（Fib）<1.5 g/L或纤维蛋白原降解产物（FDP）>20 mg/L[23]–[24]。

4. CD34⁻/HLA-DR⁻免疫表型：将患者初诊时的骨髓或外周血样本进行流式细胞术免疫分型检测，原始细胞CD34和HLA-DR均表达阴性则定义为CD34⁻/HLA-DR⁻表型，即CD34表达率<10％，HLA-DR表达率<20％。

5. 诱导治疗方案：包括IA（“7+3”）方案（标准剂量阿糖胞苷+去甲氧柔红霉素），维奈克拉（Venetoclax, VEN）联合去甲基化药物（HMA）方案，预激方案HAAG（高三尖杉酯碱、阿克拉霉素、阿糖胞苷、G-CSF）或IAG（去甲氧柔红霉素、阿糖胞苷、G-CSF）。

6. 统计学处理：连续性变量采用非参数秩和检验，分类变量采用卡方检验或Fisher精确检验比较ED与非早期死亡（non-ED）患者的基线特征。应用Logistic回归模型分析各因素对ED的影响。*P*<0.05为差异有统计学意义。纳入的因素包括年龄，体能状态（ECOG评分），查尔森合并症指数（Charlson comorbidity index, CCI），诊断时白细胞计数、血小板计数和骨髓原始细胞比例，是否CD34⁻/HLA-DR⁻表型，FLT3-ITD、DNMT3A、TET2和IDH2突变，从诊断到治疗的时间，凝血功能异常，诱导化疗方案（IA、VEN+HMA或预激方案），是否重症感染。在分析ED的危险因素时采用了VIF检验法来衡量变量间多重共线性的严重程度。以上统计学处理应用SAS 9.4和GraphPad Prism 10.0软件进行统计分析。

## 结果

1. 患者的临床特征：本研究纳入初治的NPM1^mut^ AML患者405例，男177例（43.7％），女228例（56.3％），男∶女为0.78∶1，中位年龄53（14～86）岁。ED患者35例（8.6％），死因包括重症感染14例（40.0％）、脑出血11例（31.4％）、脑梗死4例（11.4％）、肺出血3例（8.6％）、急性心肌梗死1例（2.9％）、恶性心律失常1例（2.9％），以及肿瘤溶解综合征1例（2.9％）。与non-ED患者相比，ED患者年龄较大（*P*<0.001），诊断时血小板计数较低（*P*＝0.012），骨髓原始细胞比例较高（*P*＝0.002）。值得注意的是，ED患者中凝血功能异常的发生率显著高于non-ED患者，其中PT、APTT、Fib以及FDP异常的发生率均显著高于non-ED患者（APTT：*P*＝0.010，余均*P*<0.001）；并且，流式细胞术免疫分型中原始细胞CD34⁻/HLA-DR⁻表型，也被定义为“APL-like”NPM1^mut^ AML的患者比例明显较高（*P*<0.001）。同时，ED组患者的体能状态较差，且CCI较高（*P*<0.001）。此外，ED患者中重症感染（感染性休克或呼吸衰竭）患者的比例较高（*P*<0.001）。两组患者2017年和2022年ELN危险分层的差异无统计学意义。ED患者中有10例（28.6％）患者未接受诱导化疗即死亡，两组患者的诱导方案［IA（“7+3”）、VEN联合HMA、预激方案（HAAG或IAG）］差异无统计学意义（*P*＝0.721）。non-ED患者中有176例（47.6％）患者后续接受了异基因造血干细胞移植。患者临床和实验室资料比较详见表1。

**表1:** 405例伴NPM1突变急性髓系白血病患者临床特征及基线资料

特征	早期死亡 （35例）	非早期死亡 （370例）	*P*值
性别（例，男/女）	14/21	163/207	0.644
年龄［（岁，*M*（范围）］	65（18～83）	53（14～86）	<0.001
WBC［×10^9^/L，*M*（范围）］	61.8（1.6~305.2）	43.7（0.5~366.6）	0.261
HGB［g/L，*M*（范围）］	82（30~129）	81（36~139）	0.380
PLT［×10^9^/L，*M*（范围）］	42（3~177）	56（2~448）	0.012
LDH［U/L，*M*（范围）］	602（180~7 350）	483（96~12 053）	0.133
凝血异常［例（％）］	28（80.0）	102（27.5）	<0.001
PT延长>3 s	10（28.6）	16（4.3）	<0.001
APTT延长>10 s	7（20.0）	24（6.5）	0.010
Fib<1.5 g/L	10（28.6）	25（6.8）	<0.001
FDP>20 mg/L	19（54.3）	74（20.0）	<0.001
骨髓原始细胞［％，*M*（范围）］	84（18~96）	69.5（10~97）	0.002
CD34⁻/HLA-DR⁻［例（％）］	25（71.4）	111（30.0）	<0.001
ELN 2017危险分层［例（％）］	1.000
良好	26（74.3）	271（73.2）	
中等	9（25.7）	96（25.9）	
差	0（0）	3（0.8）	
ELN 2022危险分层［例（％）］	0.161
良好	12（34.3）	185（50.0）	
中等	23（65.7）	182（49.1）	
差	0（0）	3（0.8）	
感染［例（％）］	
粒细胞缺乏伴发热	33（94.2）	309（83.5）	0.093
感染性休克或呼衰	18（51.4）	15（4.1）	<0.001
诱导化疗方案［例（％）］	0.721
IA（“7+3”）	8/25（32.0）	134（36.2）	
VEN+HMA	7/25（28.0）	117（31.6）	
预激方案	10/25（40.0）	119（32.2）	
ECOG评分［例（％）］			<0.001
≤2分	18（51.4）	318（85.9）	
>2分	17（48.6）	52（14.1）	
CCI［例（％）］			<0.001
≤3分	23（65.7）	360（97.3）	
>3分	12（34.3）	10（2.7）	

**注** LDH：乳酸脱氢酶；PT：凝血酶原时间；APTT：活化部分凝血活酶时间；Fib：纤维蛋白原；FDP：纤维蛋白原降解产物；ELN：欧洲白血病网；呼衰：呼吸衰竭；IA：去甲氧柔红霉素+阿糖胞苷；VEN：维奈克拉；HMA：去甲基化药物；ECOG：美国东部肿瘤协作组；CCI：查尔森合并症指数

2. ED和non-ED患者诊断时的基因突变图谱：405例NPM1^mut^ AML患者诊断时常见的突变为DNMT3A（51.8％）、FLT3-ITD（51.3％）、FLT3-TKD（25.1％）、IDH2（21.5％）和TET2（21.0％）突变。两组患者的基因突变图谱见图1，与non-ED患者相比，ED患者的TET2突变发生率较高（40.0％对19.1％，*P*＝0.004），FLT3-ITD突变发生率较高，但差异无统计学意义（65.7％对50.0％，*P*＝0.075）。同时，ED患者IDH2突变发生率较低（8.5％对22.7％，*P*＝0.052）。

**图1:**
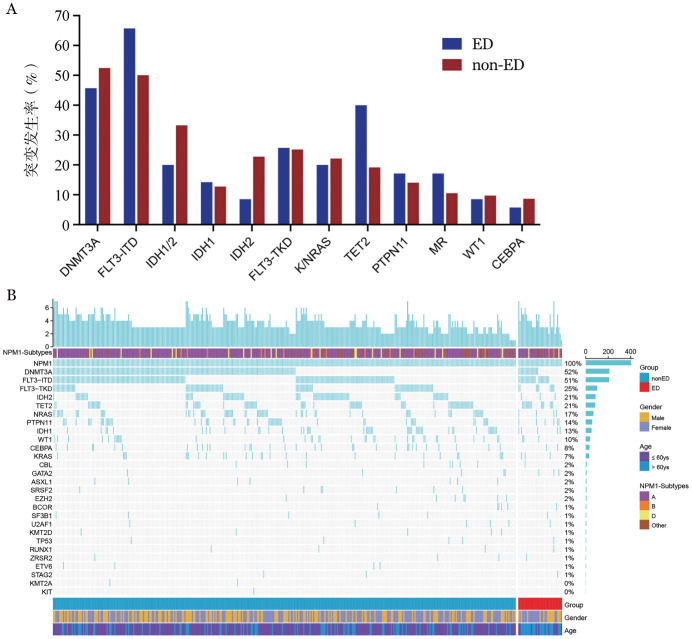
405例伴NPM1突变急性髓系白血病患者的基因突变谱 **A** 早期死亡（ED）和非早期死亡（non-ED）患者基因突变率比较；**B** 所有患者基因突变谱

3. 影响ED的危险因素：根据患者的年龄和诊断时血小板计数分别绘制ROC曲线，通过ROC曲线下面积的约登指数得到年龄的cutoff值为60岁，血小板计数的cutoff值为20×10^9^/L。单因素Logistic回归分析结果显示，高龄、ECOG评分、CCI、低血小板计数、高骨髓原始细胞比例、凝血功能异常、CD34⁻/HLA-DR⁻表型、TET2突变以及重症感染是ED的危险因素。将单因素分析中*P*<0.1的因素纳入多因素Logistic回归分析，结果显示CCI、低血小板计数、凝血功能异常、CD34⁻/HLA-DR⁻表型和重症感染是ED的独立危险因素（表2）。

**表2:** 影响伴NPM1突变急性髓系白血病患者早期死亡的单因素及多因素分析

变量	单因素分析			多因素分析		
	*OR*	95％*CI*	*P*值	*OR*	95％*CI*	*P*值
年龄>60岁	5.470	2.667～11.770	<0.001	1.432	0.355～5.639	0.607
ECOG评分≥2分	5.776	2.784～11.980	<0.001	1.547	0.381～6.187	0.535
CCI>3分	18.780	7.377～49.150	<0.001	10.540	2.289～55.840	0.003
WBC≥100×10^9^/L	0.926	0.379～2.040	0.855	–	–	–
PLT≤20×10^9^/L	4.621	1.955～10.350	<0.001	10.980	2.598～49.790	0.001
凝血功能异常	10.510	4.692～26.820	<0.001	7.126	1.967～31.410	0.005
骨髓原始细胞>50％	3.797	1.461～12.980	0.014	6.021	1.208～46.290	0.051
CD34⁻/HLA-DR⁻	5.833	2.788～13.120	<0.001	6.416	2.054～23.410	0.002
FLT3-ITD突变	1.917	0.942～4.089	0.080	0.702	0.218～2.247	0.547
DNMT3A突变	0.764	0.377～1.531	0.448	–	–	–
TET2突变	2.808	1.336～5.750	0.005	1.130	0.308～3.935	0.849
IDH2突变	0.319	0.075～0.921	0.064	0.126	0.020～0.593	0.016
诊断到治疗的时间	0.993	0.903～1.076	0.878	–	–	–
诱导化疗方案			
IA方案	0.528	0.219～1.145	0.126	–	–	–
VEN+HMA方案	0.541	0.212～1.208	0.159	–	–	–
预激方案	0.844	0.376～1.764	0.663	–	–	–
重症感染	25.060	10.940～59.320	<0.001	59.080	15.820～274.900	<0.001

**注** ECOG：美国东部肿瘤协作组；CCI：查尔森合并症指数；IA：去甲氧柔红霉素+阿糖胞苷；VEN：维奈克拉；HMA：去甲基化药物；–：未纳入多因素分析

## 讨论

我们的研究首次探讨了NPM1^mut^ AML患者ED相关的因素，除了众所周知的重症感染外，我们发现共患病指数CCI、低血小板计数、凝血功能异常、CD34⁻/HLA-DR⁻表型和重症感染是ED的独立危险因素。

有研究者报道共患病指数CCI评分与AML及APL的ED相关[25]–[27]。我们的研究中，单因素及多因素分析提示CCI也是NPM1^mut^ AML患者ED的独立危险因素，CCI评分越高，患者的ED风险越高，与文献报道一致。

出血是AML患者ED的主要原因之一，尤其是脑、消化道等重要脏器的出血，而低血小板计数和凝血功能异常是出血的重要原因，与我们的研究结果一致。同时，有研究发现，凝血功能异常可能与“APL-like”NPM1^mut^ AML具有相关性，“APL-like”表型即CD34⁻/HLA-DR⁻免疫表型，增加了脏器出血和ED的风险[14]–[18]。另外，有研究发现这类患者骨髓原始细胞含有大量嗜天青颗粒，而这一特征通常被认为是APL的形态学特征，并且该类患者的Fib、D-二聚体水平和出血事件的发生率与APL患者相当[14]。在我们的队列中，观察到ED患者中CD34⁻/HLA-DR⁻表型的患者比例显著高于non-ED患者（71.4％对30.0％，*P*<0.001），以及ED患者中凝血功能异常的发生率显著高于non-ED患者；并且，CD34⁻/HLA-DR⁻表型的患者中凝血功能异常的患者比例显著高于非CD34⁻/HLA-DR⁻表型的患者（49.3％对23.4％，*P*<0.001）。因此，我们推测NPM1^mut^ AML患者的凝血功能障碍及ED可能也与“APL-like”这一亚类有关。

同时，有研究者根据流式细胞术结果将CD34和HLA-DR双阴性表型定义为“APL-like”NPM1^mut^ AML，并比较了“APL-like”和“non-APL-like”NPM1^mut^ AML患者之间的分子遗传学差异，观察到“APL-like”NPM1^mut^ AML患者IDH1/2和TET2突变的发生率较高，DNMT3A突变发生率较低，RAS通路和FLT3突变的发生率没有差异[15],[19]–[20]。而我们的研究也发现ED患者的TET2突变的发生率较高，与文献报道一致[15],[19]–[20]，但IDH1/2和DNMT3A突变发生率差异无统计学意义，甚至IDH2的发生率较低，需设计前瞻性临床研究来进一步验证。有趣的是，我们多因素分析结果显示IDH2突变是ED的独立保护因素，可能是因为IDH2突变NPM1^mut^ AML患者的生存优于IDH2野生型患者[8]，但仍需更大规模的多中心回顾性研究或前瞻性多中心临床试验来评估IDH2突变对ED的预测价值。

综上所述，重症感染和出血是NPM1^mut^ AML患者ED的主要危险因素，而CD34⁻/HLA-DR⁻免疫表型增加了NPM1^mut^ AML患者出血和ED的风险，尽管“APL-like”NPM1^mut^ AML患者的出血和ED风险高，但有文献报道该类患者的长期OS和EFS/RFS与“non-APL-like”NPM1^mut^ AML患者相当，甚至优于“non-APL-like”NPM1^mut^ AML患者[15],[19]–[20]。因此，我们应该更加积极处理这类患者的凝血功能障碍和注意预防出血事件的发生，同时，“APL-like”NPM1^mut^ AML引起凝血功能异常以及ED的机制和治疗仍有待进一步探索。
